# Serology survey of chikungunya virus in high-risk pregnant women and placental tissue findings

**DOI:** 10.1590/S1678-9946202567043

**Published:** 2025-07-07

**Authors:** Thamirys Cosmo Grillo Fajardo, Antonio Carlos de Quadros, Clóvis Antonio Lopes Pinto, Geovane Ribeiro Dos Santos, Andrea Cristina Botelho da Silva, Daniel Thome Catalan, Ana Paula Antunes Pascalicchio Bertozzi, Rosa Estela Gazeta, Antonio Fernandes Moron, Saulo Duarte Passos, Jundiai Zika Cohort Group, Alexandra Siqueira Mello, Antoni Soriano-Arandes, Ana Alarcon, Alfredo Garcia-Alix, Alify Bertoldo da Silva, Dora Fix Ventura, Danielle Bruna Leal de Oliveira, Danilla Soares Tambalo, Diego da Silva Lima, Dirce Takako Fujiwara, Edison Luiz Durigon, Eduardo Roberto Bagne, Fernanda Guerra Velasco, Luiz Claudio Portnoi Baran, Fernando Novo Arita, Francisco Del Moral Hernandez, Juliana Paula Gomes de Almeida, Karen Richter Camandulli, Lucas Pires de Castro, Márcia Borges Machado, Mayana Zatz, Maria Manoela Duarte Rodrigues, Maria de Fátima Valente Rizzo, Maria Amélia Farrão, Mirella Nayane Barbosa Leite, Nemésio Florence, Patrícia Carvalho Loiola, Raquel Prestes, Rita de Cássia Aguirre Dezena, Sandra Helena Alves Bonon, Sergio Vranjac, Sérgio Rosemberg, Stephanno Gomes Pereira Sarmento, Steven Sol Witkinchi, Tathiana Ghisi de Souza, Viviane Cristina Martori Pandini, Viviam Paschoarelli Paiva

**Affiliations:** 1 Faculdade de Medicina de Jundiaí, Laboratório de Infectologia Pediátrica, Jundiaí, São Paulo, Brazil; 2 Faculdade de Medicina de Jundiaí, Departamento de Morfologia e Patologia Básica, Jundiaí, São Paulo, Brazil; 3 A.C. Camargo Câncer Center, Departamento de Patologia, São Paulo, São Paulo, Brazil; 4 Faculdade de Medicina de Jundiaí, Departamento de Pediatria, Jundiaí, São Paulo, Brazil; 5 Universidade Federal de São Paulo, Escola Paulista de Medicina, Departamento de Obstetrícia, São Paulo, São Paulo, Brazil; 6 Hospital e Maternidade Santa Joana, Departamento de Medicina Fetal, São Paulo, São Paulo, Brazil; 7 Faculdade de Medicina de Jundiaí, Departamento de Pediatria, Ambulatório de Assistência, Ensino e Pesquisa, Jundiaí, São Paulo, Brazil

**Keywords:** Arboviruses, Chikungunya virus, Placenta, Pregnancy, Serologic tests

## Abstract

Evidence suggests a risk of maternal transmission of chikungunya virus (CHIKV) during the first and third trimesters, potentially leading to miscarriage or neurological consequences for the fetus. This study aimed to conduct a serological survey for CHIKV among women with high-risk pregnancies and analyze neonatal variables and placental tissue alterations. From March 2016 to April 2021, serological, histological, and molecular tests for CHIKV were performed. Blood samples were analyzed for anti-CHIKV IgG and IgM antibodies, and placental tissue was examined for CHIKV RNA and histological changes. Among pregnant patients, 1.33% (7/526) had reactive IgG, and 0.38% (2/526) had IgM/IgG-type antibodies during delivery. Although placental histology of CHIKV disease showed alterations, no viral genetic material was identified in the analyzed tissues. Therefore, further research is needed, including the use of complementary diagnostic techniques, to better understand the impact of this relatively new disease among high-risk pregnant women and newborns.

## INTRODUCTION

Dengue, Zika, and chikungunya viruses have caused drastic socioeconomic impacts, particularly in low- and middle-income countries^1^. In 2013, chikungunya virus (CHIKV) was reported for the first time in the Americas^2^. By 2014, Brazil, Colombia, and Venezuela had the highest number of CHIKV cases in Latin America, with individuals with pre-existing comorbidities, older adults, and newborns being particularly vulnerable to infection. These arboviruses continue to pose a significant challenge, especially in underdeveloped countries like Brazil^1,3^.

According to data from the Brazilian Ministry of Health, 233,225 probable cases of chikungunya were reported between epidemiological weeks 1 and 26 of 2024. Sao Paulo State had the third-highest incidence rate in Southeastern Brazil^4^.

Globally, 90% of pregnant women reside in areas susceptible to arbovirus infection. Most of these areas are in low-resource settings, where women face significant challenges in receiving proper differential diagnosis. While some arboviruses have been well documented, many, including CHIKV, remain relatively understudied^5^.

Miscarriages have been reported among pregnant women infected with CHIKV before 16 weeks of gestation, with laboratory confirmation of the viral genome in pregnancy-related fluids and tissues, such as amniotic fluid and placenta^6,7^. Low birth weight, premature rupture of membranes, and decreased intrauterine fetal movements have also been reported^8,9^.

Congenital CHIKV infection is rare, primarily due to the placenta, which is considered a highly effective barrier against fetal infection during gestation. However, the virus can alter placental tissue structure, potentially impairing nutrient and gas exchange. This may compromise the delivery of these substances to the fetus, contributing to adverse outcomes such as preterm birth and low birth weight^10,11^.

To date, the role of CHIKV in the development of gestational complications remains unclear^12-14^. Therefore, this study aimed to conduct a serology survey of high-risk, primarily asymptomatic pregnant women in a region where the disease was not yet endemic, investigating potential associations between CHIKV infection and potential gestational and perinatal outcomes, as well as placental tissue alterations.

## MATERIALS AND METHODS

### Study design, study population, and ethics statement

This was a prospective, analytical study using a convenience sample of pregnant women enrolled in the thematic project “Vertical infection by the Zika virus and its repercussions in the maternal-infant field.”

The study was conducted from March 2016 to April 2021 and involved high-risk pregnant women referred to prenatal care at the Women’s Health Outpatient Clinic of the Jundiai University Hospital, part of the Health Promotion Management Unit in Jundiai, Sao Paulo State, Brazil.

Inclusion criteria were high-risk pregnancy and participation (mother and newborn) in the Zika virus project, with serum or plasma samples collected during both prenatal care and delivery. All participants provided written informed consent. The Jundiai Zika Cohort profile is described in Sanchez Clemente *et al*.^15^.

This study was approved by the Research Ethics Committee of the Jundiai Medical School (CAAE Nº 61349716.9.0000.5412). All procedures followed the principles outlined in the Declaration of Helsinki (1964, as revised in 1975, 1983, 1989, 1996, and 2000). Written informed consent was obtained from all participants.

**Figure 1:**
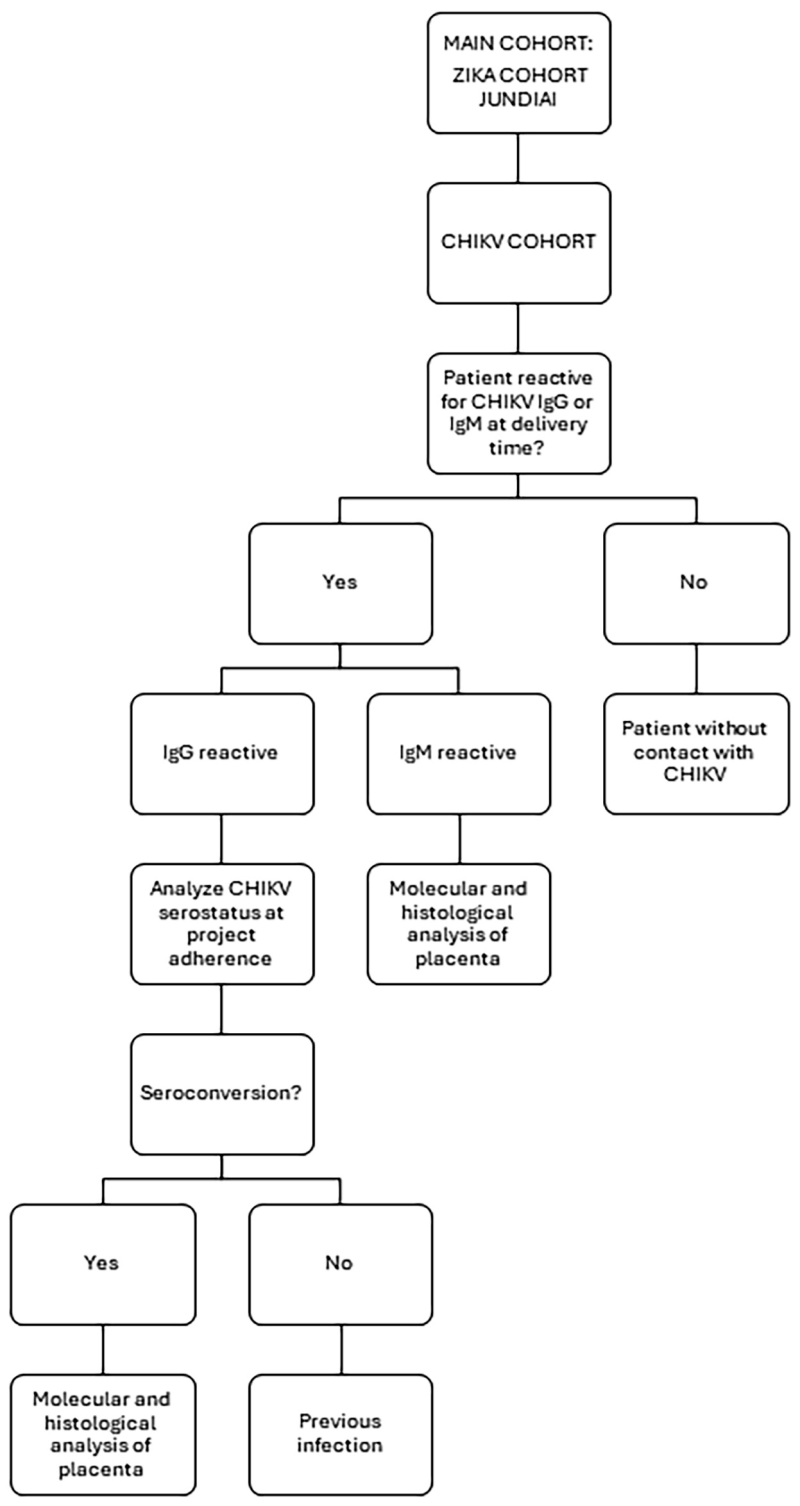
Flowchart of participants during clinical trial.

Maternal serum or plasma samples collected from peripheral whole blood at delivery were analyzed for anti-CHIKV IgG and IgM using enzyme-linked immunosorbent assays (ELISA) to determine seroincidence and seroprevalence in 526 pregnant women (n = 526). Women with reactive IgM alone or both IgM and IgG at delivery were classified as having acute infection. For women with reactive IgG only at delivery, additional ELISA testing was performed on samples collected at enrollment to evaluate seroconversion. Seroconversion during gestation was defined using the following criteria, requiring a minimum interval of 10 days between enrollment and delivery: (a) a four-fold increase in anti-CHIKV IgG antibody titers; (b) non-reactive anti-CHIKV antibodies at enrollment and reactive IgM and/or IgG at delivery; and (c) reactive IgM at enrollment with subsequent reactive IgG at delivery. Placental tissue was analyzed histologically and molecularly only in cases classified as acute infection or seroconversion (Figure 1). Perinatal outcomes were also assessed specifically for acute infection and seroconversion.

### Analytical methods

Biological serum or plasma samples were subjected to ELISAs to detect anti-CHIKV IgG and IgM antibodies. Commercial anti-CHIKV ELISA IgG and IgM kits (Euroimmun^®^, Lübeck, Germany) were used following the manufacturer’s instructions. Placental materials in paraffin slides were first extracted with xylol, followed by serial baths of alcohol and RNAse-free water. Genetic material was then extracted using the TRIzol^®^ reagent (ThermoFisher Scientific, Bremen, Germany) following the manufacturer’s instructions. For molecular analysis, XGEN MULTI ZDC (XGEN^®^, Mobius Life Technology, Brazil) was used, also following the manufacturer’s instructions. Histological analysis of the placentas was performed by the Department of Morphology and Pathology at Jundiai Medical School.

### Statistical analysis

Proportions of seropositive individuals are presented as percentages. The mid-p exact test, a non-parametric method, was used for cross-tabulation of categorical variables. Statistical significance was set at p <0.05, with a 95% confidence interval. Odds ratios were calculated for each variable. OpenEpi software was used to generate 2x2 contingency tables for the analysis.

## RESULTS

This study included 526 pregnant women. Regarding maternal seroprevalence at delivery, 1.33% (7/526) tested positive for anti-CHIKV IgG, and 0.38% (2/526) for both anti-CHIKV IgM and IgG. No women were positive for IgM alone during this period.

For patients with reactive IgM/IgG at delivery, additional maternal and neonatal laboratory tests were performed. These results are described in the study by Fajardo *et al*.^16^.

The average age of the women was 28 years (range: 13–47 years). The average age of women with reactive anti-CHIKV antibodies was 26 years (median not provided), while those with non-reactive anti-CHIKV results had an average age of 28.3 years (median 28 years). The mean gestational age at delivery was 38 weeks and three days (median 38.4 weeks), ranging from 28 weeks to 44 weeks and six days. In total, 77 women had preterm deliveries, including one with reactive anti-CHIKV IgG antibodies. Of the 22 women with post-term deliveries, one was reactive for anti-CHIKV IgG. Most participants resided in Jundiai (71.4%), identified as white (53.6%), lived in brick houses (99.3%), and had completed high school (44%).

Fifty-five distinct etiologies requiring prenatal monitoring were identified within the studied population. Furthermore, 193 pregnant women (36.7%) exhibited at least two of these conditions requiring outpatient care. Systemic arterial hypertension and diabetes mellitus (gestational, type I, and type II) constituted 54.6% of the most prevalent etiologies. These were followed by advanced maternal age (22.24%), adolescent pregnancy (13.31%), thyroid dysfunction (4.20%), and obesity (0.4%).

Of the patients who tested positive for anti-CHIKV IgG or IgG/IgM antibodies at delivery, only one presented with symptoms suggestive of arboviral infection. Therefore, approximately 89% of these patients were asymptomatic. Statistical analysis suggested a significant association between obesity and CHIKV infection among high-risk pregnant women (p = 0.0342). Women with obesity exhibited a slightly higher prevalence (3%) of CHIKV infection compared to those with other comorbidities. Due to the limited number of cases observed, further studies are needed to investigate this subgroup more thoroughly.

Among patients with reactive anti-CHIKV IgG results at the time of delivery, four showed characteristics of seroconversion during pregnancy. Of these, two occurred between the second and third trimesters, one during the third trimester, and one between the first and third trimesters. Serological characteristics and other relevant patient data are detailed in Table 1.

**Table 1:** Serological characteristics and relevant data for patients with reactive IgG and IgM for CHIKV

Case	Age	Risk factor(s)	Arbovirus infection symptoms	Adhesion period		GE	Trimester	Delivery period		GE	Seroconvertion/Acute infection
				IgM	IgG			IgM	IgG		
22	28	Syphilis, hypothyroidism, twin pregnancy, uterine growth restriction (UGR)	No	Non-reactive	Non-reactive	20	2º	Non-reactive	Reactive	29º	Yes
52	17	Adolescent, Obesity, Rh(-), UTI	No	Non-reactive	Reactive	16	2º	Non-reactive	Reactive	38º	No
238	30	Hypertension, Previous abortion	No	Non-reactive	Reactive	27	3º	Non-reactive	Reactive	38º	No
319	28	Type 1 diabetes mellitus, Hypertension, Obesity	No	Non-reactive	Non-reactive	27	3º	Non-reactive	Reactive	44º	Yes
354	24	Gestational diabetes, UTI	No	Non-reactive	Non-reactive	24	2º	Non-reactive	Reactive	40º	Yes
468	24	Zika infection	Yes	Reactive	Reactive	30	3º	Reactive	Reactive	40º	Yes
471	36	Myomectomy	No	Non-reactive	Reactive	31	3º	Non-reactive	Reactive	36º	No
585	23	Deafness	No	Reactive	Reactive	36	3º	Reactive	Reactive	37º	Yes
756	21	Pre-eclampsia, obesity, Zika infection	No	Reactive	Reactive	9	1º	Non-reactive	Reactive	37º	Yes

GE = gestational period (weeks); IgM = IgM immunoglobulin; IgG = IgG immunoglobulin; UTI = urinary tract infection.

For women with acute infection and seroconversion, clinical data were collected for their respective newborns, and perinatal outcomes were documented for each case. Histological analysis of placental tissue was also performed, along with RT-PCR testing for DENV, ZIKV, and CHIKV. Detailed case descriptions are presented in Table 2.

**Table 2:** Perinatal outcomes and placental findings among cases of gestational CHIKV

Case	Birth gestational age (weeks/ days)	Placental histopathology	RT-PCR Result	Mother with arbovirus infection symptoms	Childbirth	Weight (g)	Weight percentile	Classifi- cation	Length (cm)	Z-Score (Length)	HC (cm)	Z- Score (HC)	APGAR 5	IUGR
022 (N1)	29/2	Syphilis, hypothyroidism, twin pregnancy, uterine growth restriction (UGR)	Not detected	No	Natural	915	4.97	SGA	34	-1.8955	26	-0.6623	7	Yes
022 (N2)						1,355	75.2	AGA	38	-0.1928	28	0.7809	10	Yes
319	44/6	Intervillous fibrin, cord edema, chorioamnionitis, and perivillous fibrin and calcification	Not detected	No	Cesarean Section	3,915	97.49	LGA	48	-0.2422	37	2.9376	9	No
354	40/0	Delayed maturation, Fibrin and perivillous calcification, cord edema, ground glass cells	Not detected	No	Natural	3,740	87.4	AGA	51,5	1.4499	35	1.1173	9	No
468	40/0	Fibrin deposition and intervillous calcification	Not detected	Yes	Cesarean Section	4,205	98.3	LGA	52	1.7508	37	2.6803	9	No
585	37/2	Fibrin deposition and calcification in intervillus and infarct areas	Not detected	No	Natural	3,280	85.48		48	0.3082	31	-1.5034	9	No
756	37/6	Without abnormalities	Not detected	No	Natural	3,940	97.54	LGA	52	1.9275	37	2.4003	8	No

RT-PCR = polymerase chain reaction for Zika, dengue and Chikungunya viruses; % = Percentile; HC = head circumference; APGAR = 5 min; IUGR = intrauterine growth restriction; SGA = small for gestational age; AGA = suitable for gestational age; LGA = large for gestational age.

## DISCUSSION

Recent data from the Ministry of Health’s epidemiological bulletin indicate that, between January and July 2024, the Southeast region had the third-highest number of suspected CHIKV cases and the highest incidence rate, demonstrating the virus’s rapid spread to other Brazilian states and its potential for dissemination^4^.

Despite the growing body of research on CHIKV, inconsistent results persist, largely due to variations in geographic regions, study periods, clinical case definitions, and differences in the sex and age distribution of affected populations. Furthermore, methodological diversity of seroepidemiological studies contributes to this discrepancy^12-14^.

Regarding the maternal serological survey, 1.33% of the women tested positive for anti-CHIKV IgG, and 0.38% for both anti-CHIKV IgM/IgG. However, this study involved a specific population—high-risk pregnant women in a non-endemic region—most of whom were asymptomatic. This unique combination of factors limits direct comparison with other published studies.

Two clinical observations merit attention. First, although not statistically significant, most women with reactive anti-CHIKV results were asymptomatic, which contrasts with previous research reporting symptomatic CHIKV infection in approximately 80% to 97% of patients^17^. These findings raise the question of whether high-risk pregnancy may influence the manifestation of CHIKV symptoms compared to non-high-risk pregnancies. While no studies have specifically addressed this hypothesis, Foeller *et al*.^18^ reported that pregnancy may confer a protective effect by reducing symptom duration and mitigating long-term sequelae of CHIKV infection. Second, obesity was significantly associated with CHIKV infection in high-risk pregnant women (p = 0.0342), with a slightly higher prevalence (3%) of infection observed in women with obesity compared to those with other comorbidities. However, this finding should be interpreted cautiously given the limited sample size, and further research is needed.

Moreover, pregnant women with comorbidities are more likely to exhibit placental changes^19^. In this study, histopathological analysis of placental tissue from women with CHIKV seroconversion or infection revealed frequent perivillous and intervillous fibrin deposition and areas of calcification. However, these alterations are consistent with placental changes associated with the underlying pathologies present in high-risk pregnancies^19^.

Placental lesions attributable to CHIKV are rare, as is the detection of viral genetic material in placental tissue^19^. In this study, despite serological evidence of seroconversion or active infection, CHIKV RNA was not detected in placental samples, a finding consistent with those of Platt *et al*.^20^. Furthermore, no statistically significant perinatal outcomes associated with CHIKV infection were observed. This may be related to the timing of CHIKV infection in the women studied, which occurred predominantly in the third trimester. Beyond the gestational period, placental tissue may be a less permissive environment for CHIKV replication, and potentially other flaviviruses, as suggested by in vitro and animal model studies^20^.

## CONCLUSION

Although limited, the findings of this study underscore the need for further investigation into the impact of CHIKV infection on high-risk pregnancies, particularly in non-endemic regions. The limited sample size and lack of a robust control group restrict the generalizability of these findings. Therefore, prospective studies with larger cohorts and longitudinal follow-up are recommended. Furthermore, investigating other biological markers, such as those assessed via immunohistochemistry, and analyzing inflammatory biomarkers may further elucidate the pathogenic mechanisms involved.
